# The lived experience of Omani adolescents and young adults with mental illness: A qualitative study

**DOI:** 10.1371/journal.pone.0294856

**Published:** 2023-11-27

**Authors:** Omar Al Omari, Gerald Amandu, Samir Al-Adawi, Zubaida Shebani, Ibtisam Al Harthy, Arwa Obeidat, Khloud Al Dameery, Mohammad Al Qadire, Iman Al Hashmi, Abduallh Al Khawldeh, Mohammed ALBashtawy, Maen Aljezawi

**Affiliations:** 1 College of Nursing, Sultan Qaboos University, Muscat, Oman; 2 College of Nursing, Yarmouk University, Irbid, Jordan; 3 College of Medicine and Health Sciences, Sultan Qaboos University, Muscat, Oman; 4 College of Education, Sultan Qaboos University, Muscat, Oman; 5 Princess Salma Faculty of Nursing Al al-Bayt University, Mafraq, Jordan; School of Health Binawan: Universitas Binawan, INDONESIA

## Abstract

There is currently limited knowledge about the firsthand experiences of adolescents and young adults with mental health problems and the meanings they ascribe to these experiences, particularly within Arab countries. This study, therefore, aimed to explore the lived experience of Omani adolescents and young adults with a mental health problem. A sample of 15 participants aged 13–22 diagnosed with a range of mental health problems took part in the study. A qualitative interview guide consisting of open-ended questions was used to allow participants to speak in-depth about their experiences. Using the thematic analysis approach to uncover patterns in the data, three major themes emerged: “living in darkness”, “perilous journey” and “uncertain future”. Results show that the progress of adolescents and young adults with mental health problems is characterized by several challenges; the most significant of which is having insufficient knowledge about their illness, leading to unnecessary delays in their treatment. These findings shed light on the breadth and depth of the experience of adolescents and young adults with mental health problems and lay the groundwork for further examinations. Implications lie in the development of approaches for preventing or mitigating difficulties faced by adolescents and young adults with mental health problems.

## Introduction

Mental health problems among adolescents and young adults (AYA) have surged significantly during recent decades [1–3]. Globally, it has been estimated that one out of every seven AYA are vulnerable to developing mental health problems [4]. The escalated trends in mental health problem prevalence presents an immediate public health challenge deserving urgent concern [1]. That is the magnitude of this problem, which impacts many AYA.

This challenge is further compounded by the devastating implications. Mental health problems are becoming more widespread, and more people require to visit counseling and psychiatric centers. Additionally, the dependence upon psychotropic agents to treat this group of AYA presents another complex dimension of care. [5, 6]. It has also been reported that the presence of mental health problems among AYA has the potential to affect their education and ability to acquire important life skills which, in turn, could have an effect on their quality of life and meaningful existence [7].

One way to shed light on the challenges faced by AYA with mental health problems is to employ a lived experience paradigm [8] in examining their subjective experience. The lived experience paradigm is part of a phenomenological or ‘emic’ approach and, according to Neubauer, Witkop [8], the paradigm aims to understand “*what* was experienced and *how* it was experienced” (p. 91). While a plethora of studies have employed the lived experience paradigm in developed countries such as the United States, Canada, the United Kingdom and other countries in Western Europe as well as some countries on the Pacific Rim, there have been very few investigations using the paradigm in emerging economies [9]. Demographic trends in developing countries suggest the presence of a ‘youth bulge’ in the pyramidal population structure, indicating the preponderance of young people [10]. More initiatives using emic approaches are therefore needed to understand the experience of AYA suffering from mental health problems in developing countries so that mechanisms to safeguard their well-being could be developed.

Studies carried out in Western countries and using phenomenological approaches such as lived experience have suggested that AYA with mental health problems go through unpleasant experiences in their search for treatment [11, 12], with negative repercussions on their quest for a meaningful existence [13]. In a study by Woodgate [14], adolescents with mental health problems described themselves as “walking zombies”, meaning that they saw themselves devoid of free will and the ability to control their lives. Additionally, previous research has linked the presence of mental health problems among AYA with poor academic performance [3, 15] as well as inadequate self-care, poor personal development, and poor socialization skills [16, 17]. Researchers have documented that AYA with mental health problems have an increased chance of causing self-harm [18] as well as developing sexual and reproductive health problems [19]. Such detrimental and maladaptive behaviour has been reported to persist even when there is remission of overt mental health problems [14, 20]. In most societies around the world, there are varying degrees of exclusion and marginalization of people with mental health problems and indeed AYA experience stigma and discrimination. This is consistent with reports that mental health problems among AYA are intransigent and debilitating and often create dependency [4].

The Arabian Gulf countries are “home to one of the youngest populations in the world” [21]. As one out of seven people globally is likely to succumb to mental health problems [4], it is likely that such a trend also exists in the Gulf countries, although as always, mental health problems often cause different degrees of distress and symptomatology [22]. While the standard of living in this region has significantly improved in recent years due to the exploitation of hydrocarbon and the development of transhipment services [23], the welfare of people with mental health problems is often not given enough attention, likely due to a lack of mental health literacy in the region. A systematic review on mental health literacy conducted by Elyamani, Naja [24] discovered that mental health awareness remains suboptimal in the Gulf countries among healthcare professionals and the public. Parallel to the low level of mental health literacy, studies have suggested the presence of pervasive stigma toward people with mental health problems [25, 26]. Similarly, despite the development of the biomedical model, there is wide under-utilization of existing psychiatric services [27]. Mirza, Al-Huseini [28] have indicated that due to the long duration of untreated mental health problems among AYA in Oman, many of those who eventually seek consultation from psychiatric services are likely to be those with more advanced and irreversible pathology, making them impervious to the treatment offered.

While a reasonable number of etic (outsider-oriented) studies have illuminated the prevalence of mental health problems, there remains a significant gap in understanding the emic (insider-oriented) approach, this study is distinct in its focus on Omani adolescents and young adults. That is, while prior work has been invaluable, this research acknowledges a need for a personalized approach within Oman where cultural and social factors may influence experiences of AYA with mental health problem. This study explores the lived experience of Omani AYA with mental health problems. The objective is to gain a deeper understanding of the key characteristics of their lives, with the overarching aim of identifying critical factors that may contribute to their mental health issues. Through this research, we provided an insight into the desperate requirement for more specialized mental health services for these AYA in this cultural setting.

## Methods

Interpretive phenomenological analysis (IPA) methodology was used in this study to describe the experiences of AYA with mental health problems. This approach was used because, when little is known about the phenomenon being examined, the methodology allows for complex descriptions of lived experiences to be reported and documented [29, 30]. In-depth interviews using open-ended questions were used to allow participants to express their views and experiences in their own words. Interviews were then transcribed and converted into a format suitable for analysis. Following interpretive methodology, thematic analysis was applied to identify recurring themes and meanings within the data. To understand and interpret the meaning the AYA ascribed to their experience, a systematic process was used for coding and categorizing data. This process is described in Section 2.4.

### 2.1. Sample/participants

Fifteen AYA were recruited for the study and interviewed in depth. This number is adequate for achieving the objectives of the study and is consistent with IPA research, which typically advocates a small sample size [30]. The data collection continued until theoretical saturation was achieved and no new themes, insights, or significant information emerged from subsequent interviews, ensuring that we thoroughly explored the experiences of adolescents with mental health issues. The inclusion criteria were (1) being an AYA aged between 10 and 24 years; (2) being diagnosed with a mental health problem such as bipolar disorder, borderline personality, obsessive-compulsive disorders and depression for more than nine months; (3) visiting the outpatient clinics; (4) able to provide informed consent.

### 2.2 Ethical considerations

Ethical approval was obtained from the Medical Research Ethics Committee at Sultan Qaboos University (SQU-EC/313/2020). The study followed the ethical principles outlined in the Helsinki declaration. All participants and their parents provided written informed consent. The potential participants’ contact information was obtained from the outpatient clinics where the patients were stable and able to provide consent. Any data that could revealed the identity of the participants was kept strictly confidential. The recorded interviews were saved within password-protected files on the lead investigator’ s workstation. The researchers were aware that the interview topic might elicit a negative adverse reaction from participants, and precautions were taken to ensure painful emotional reactions would not be triggered. Moreover, the research assistant who conducted these interviews is well-versed in qualitative phenomenological research and concurrently holds a master’s degree in applied psychology, with wide clinical experience in crisis intervention. This background may have helped facilitate the establishment of rapport with the participants as well as adhere to an unconditional positive stance during the interviews [31]. The psychologists were instructed that if participants experienced any emotional distress during the interview, they should take necessary actions such as providing a break and/or providing interventions to reduce their stress level. If the stress persists, the psychologists can refer the participants to designated mental health services, as treatment is provided free of charge in Oman.

### 2.3 Data collection

The data collection took place between October 30, 2021, and November 10, 2022. The research assistants requested the contact details of the parents of AYA with mental health problems from the nurse managers in the outpatient department. Parents were then contacted by telephone during which the purpose and nature of the research were explained to them, and they were asked for their consent to interview their children. After obtaining written consent from the AYA and their parents, the researchers organized face-to-face interview sessions with the AYA in the presence of their parents at a mutually agreed time and place. At the beginning of each interview, participants were presented with an information sheet to read which included information about the purpose and procedure of the study. They were given the opportunity to ask any questions related to the study. After agreeing to participate, they were required to sign a consent form. Participants were informed that their responses would be kept strictly confidential and that they were allowed to withdraw from the study at any point and without giving reason. Care was taken to ensure participants were comfortable prior to and during the interview. Breaks during the interview were encouraged, if needed. Additionally, participants were informed that if they experienced any distress or discomfort during the interview, the researcher would promptly stop the interview. Interviews were conducted by an experienced research assistant with graduate-level expertise in clinical psychology. The researcher was trained to build rapport with the interviewees by establishing trust, a positive relationship and open communication. To ensure data integrity, each independent and in-depth semi-structured interview was recorded digitally, with each interview lasting on average 60 minutes. Examples of the interview questions are presented in Table 1.

**Table 1:** Sample questions for the interview.

• Could you please tell me the story of your mental health problem? • What kind of challenges have you faced because of your mental health problem? • How do people look at you? How do you think your friends perceive you? • What responsibility does the community have towards people with mental health problems?

### 2.4 Data analysis

The data was initially gathered in the Arabic language to allow participants to convey their complete narratives. Subsequently, the interviews were translated into English by proficient bilingual experts to preserve the original meaning of the transcribed interview text. The interviews were transcribed verbatim and content analysis was applied to extract any hidden meanings in participants’ stories. The two most senior researchers working independently performed the open coding, which entails reading the content numerous times to become familiar with it and then identifying meaningful “chunks” of data termed as codes. Codes represent meaningful and significant narration of the interview transcripts. The researchers then compared the various codes with one another to establish differences and similarities between them. Codes with similar meanings were placed together in the same categories and themes. Two qualitative researchers compared these various categories and together formulated the general themes, which constituted the main findings of the present study. To ensure high quality, data analysis and interpretation were initially conducted separately, and jointly only later by the two independent qualitative researchers, both of whom have doctoral preparation.

### 2.5 Rigour

The researchers achieved rigour by observing the four classic criteria for establishing quality in qualitative research. The first principle, *credibility*, which focuses on ensuring credible findings, was attained through prolonged engagement with the participants and the data and divergent case analysis. This involved conducting in-depth interviews with each participant that lasted, on average, approximately 60 minutes. During these interviews, we employed strategies to establish rapport and trust with the participants. The second criterion of transferability, which focuses on ensuring that the findings are applicable to participants who share the Omani AYA same context, was attained by providing a rich description of participants’ responses and the researcher’s interpretations of these narratives. The researchers achieved the third criteria of *dependability* by independently reviewing the research data, arriving at codes separately, and then jointly agreeing on themes after a lengthy discussion. The two researchers re-examined the data together and discrepancies in themes or code identification were discussed until agreement was reached. While this process took a long time, it helped to improve the data’s trustworthiness. The researchers established the fourth criterion of *confirmability*, which ensures that the findings are a product of participants’ responses and not the researchers’s biases, motivations, interests, or perspectives, by supporting their interpretation of the findings with verbatim descriptions for other scholars to independently audit and evaluate.

## Results

The data was collected between 17^th^ of October 2021 to 19^th^ of July 2022. Fifteen AYA with a mean age of 17.2 years participated in the study (9 female, 6 male). Three had been diagnosed with Major Depressive Disorders (MDD) and bipolar disorder; seven had MDD and borderline personality disorder; another three had MDD and obsessive-compulsive disorder; and the last two suffered from MDD only. All the participants had lived with their mental illness for more than nine months since diagnosis. Three major themes emerged, corresponding to three phases related to the disease namely: the pre-diagnosis phase, the journey after diagnosis, and future life with mental illness. These themes are “living in darkness”, “perilous journey” and “uncertain future”.

### Theme 1- Living in darkness

The findings revealed that AYA had insufficient knowledge about mental illness. In effect, both were living in the dark, unaware of what was going on in the minds of the AYA. ‘Living in the Dark’ reflects a period that is typically an individual’s first encounter with mental problems, which are often unfamiliar to them. Many participants and their families found themselves in an unfamiliar territory and explained their symptoms as either superstition, physical ailment or spiritual causes. This theme depicts the transition from the mental health problem’s unfamiliarity to familiarity. Some participants said that their parents associated the signs and symptoms with a lack of faith, or with superstition and associated feelings of jealousy and envy. The parents’ linkage of mental illness to superstitious beliefs is well described by this participant:

…*in the beginning*, *I lost interest in my study and decided to not go to school… I simply sat at home*, *and I felt low… no matter what I did I had the same feeling…*. *I lost interest in people… my parents did not know what to do…*.*they said it’s the evil eye*, *and they sent me to the Sheikh to read the [holy] Qur’an (Participant “K”*, *16 years old*, *with depression*).

Similarly, participant “A”, reports that his family and their primary healthcare provider initially associated the onset of the symptoms of his mental illness with poor physical health. This was probably due to the nausea and weight loss he experienced, as illustrated in his description:

*I felt low and removed myself away [from friends and family] … I lost interest in life… I was tired*, *and I was silent all the time…I lost weight and became nauseated all the time… my parents sent me to the hospital… I spent two days in the hospital…*. *Doctors gave me some IV fluids*, *vitamins*, *some medication and sent me home (Participant “A”*, *16 years old*, *depression)*

Another instance of living in the dark is clearly explained by 20-year-old participant “B”. He described how when his illness began, both he and his family did not know what was happening. He recalled being unable to sleep as he experienced auditory hallucinations. As the family tried to understand his illness, their initial explanation was demonic possession:

*I was unable to sleep*, *nervous*, *aggressive*, *and I heard someone who whispered in my ears… my parents sent me to Sheikh to read Qur’an*. *The Sheikh said that I am possessed with demons*, *and that he needed to read Qur’an on me…my parents agreed with him*… *(Participant “B”*, *20 years old*, *depression and borderline personality disorder)*.

While most participants and their families experienced confusion at the onset of symptoms, the case of participant “AF” stood out. Contrary to the experience of other participants, her parents upon noticing her symptoms, directly accompanied her to see a psychiatrist. Participant “AF” associates this quick response of health-seeking behaviour to her parents’ previous experience with mental illness in the family; as she recalled:

*One day I took off my hijab and I fought with our neighbours. I shouted at them and when my parents discussed this occurrence with me, I also fought with them…Since we have a family history of mental illness, my parents sent me immediately to the psychiatrist (Participant “AF”, 17 years old, with depression and borderline personality disorder)*.

Participant “AA” lamented that she spent a long time with the symptoms before receiving a proper diagnosis. She explains that it was a relative who noticed her behaviour and first suggested that she might be suffering from a mental health problem:

*I spent a long time without treatment [5 months] …*.*there was no mention about mental illness… It was only after one of my relatives working as a nurse advised my parents to take me to the mental health service*. *My parents agreed and sent me there*… *(AA*, *18 years old*, *depression and borderline personality disorder)*.

Not all medical professionals in primary care who attended to these AYA linked their behaviour to mental health problems. In some cases, family members and professionals at school were the ones correctly associated the AYA’s behaviours with mental illness. It is only then that the affected adolescents were referred to appropriate mental health services.

### Theme 2- Perilous journey

The term ’Perilous Journey’ symbolizes the countless challenges and hurdles adolescents encountered while trying to navigate the intricate world of mental health treatment. From rushed medical appointments to therapy sessions that seemed unhelpful, these young individuals had to overcome a series of obstacles that truly tested their strength and hope. The first challenge the participants experienced was the short duration of their meetings with doctors. The majority of participants said that the psychiatrists and psychologists they saw did not provide sufficient time for consultation which limited how much of their experience they could share. In effect, many felt that the consultations did not make much difference to their lives. This is well elaborated in the description below by participant “S”:

*…I waited for this [interview] for ages and ages*, *but when it happened I was disappointed… the interview did not take a long time and made no difference*…*”(Participant “S”*, *19 years old*, *MDD and bipolar disorder)*.

Participants also felt that their doctors did not give them enough attention during consultations and seemed rushed. Unfortunately, because there is a shortage of mental health services, consultations with psychiatrists and other mental health providers are often short in order to meet demand. One of the participants, “Q”, reflecting on her experience said:

*I felt the doctors just wanted to finish [the consultation] quickly… they did not speak a lot with me*…*they only prescribed the medications without any discussion and explanations (Participant “Q”*, *20 years old*, *depression and obsessive-compulsive disorder)*.

The third finding that made the treatment journey difficult is the realization that some of the therapeutic sessions, particularly with the psychologists, were not productive. As a result, some of the AYA stopped attending sessions as they saw them as unhelpful and a waste of time:

…*we were speaking together*…, *sharing stories… she [the psychologist] would tell me to write my thoughts on paper and exercise… [but] nothing happened … I found it useless*. *After about two sessions I quit” (Participant “AM”*, *21 years old*, *depression and obsessive-compulsive disorder)*.

Another challenge that complicated the treatment journey is the discovery that many of the prescribed medications had a plethora of side effects that they had hitherto not experienced. Negative side effects included increased sleepiness and weight gain:

… *[after] I started my medication…everything changed*. *I lost my energy and I always felt tired and wanted to sleep…I put on weight which made me stop the medication*, *but my symptoms became worse than before*, *so I went back to medication (Participant “S”*, *19 years old*, *depression and bipolar disorder)*.

While the participants experienced a challenges during their treatment, a number acknowledged that their mental health had improved significantly following treatment. The hope and optimism experienced by the AYA were partly because they were able to access formal mental health services after a long period of being in the dark with their symptoms, unsure of what to do:

… *I was happy*… *at last [having waited for more than 6 months]*, *they found a solution to my problems… I hope I will be better (Participant “SS”*, *19 years old*, *depression and bipolar disorder)*.

Another reason for the optimism expressed by the AYA and their family members, despite the perilous nature of the treatment, was the realization that despite some side effects, the medications prescribed by healthcare workers helped them to control symptoms:

… *I took my medication … people in the hospital*, *medication and family helped me to control the disease… I am almost back to normal … I am very positive (Participant “YM”*, *19 years old*, *depression)*.

### Theme 3- Uncertain future

‘Uncertain future’ delves into the worries of the participants regarding what awaits them after receiving a diagnosis and undergoing treatment. Some struggled with fears of losing control over their thoughts, the possibility of becoming dependent on medication, and/ or that their disease might return. This fear of what the future holds for them is clearly illustrated in the narrative of this 18-year-old student:

…*although my thoughts did not change [as yet] and still I am thinking [well]*, *I try to resist [negative] thoughts … [I also] try to avoid interactions with my family that would not help me… I try to avoid any type of pressure*, *but I am not sure if I will continue doing this [successfully] in future” (Participant “AA”*, *18 years*, *with depression and borderline personality disorder)*.

On the other hand, some AYA feared taking medication and the possibility of becoming dependent on the prescribed medication. A closely related concern was the side effects they produced, as narrated below:

*The medication comes with many adverse effects*… *I do not know which one is more harmful*, *the negative effect of the medication or the disease itself*. *I no longer know myself*. *I do not know when I will stop the medication and what will happen if I stop it (Participant* “*SS”*, *19 years*, *with depression and bipolar disorder)*.

For some, the uncertainty about their future was associated with whether they would be able to return to school and complete their studies, which had been interrupted by their mental illness. This concern about their perceived ability to continue with their studies upon completing treatment is well-articulated in the description by this participant:

*I have missed one year of my studies now and I do not know when I will be able to resume my studies*… *(Participant “K”*, *20 years*, *borderline personality disorder)*.

These concerns about their future seem to suggest that health team members and their parents did not provide enough support to AYA about the expected prognosis of their illness and its impact on their future. Such support would have allayed their anxiety.

## Discussion

Various studies in Oman have explored the prevalence and associated factors among children and AYA with mental illness [10, 32]. To date, no qualitative studies have explored the lived experiences of Omani AYA with mental health problems. The onset of mental health problems is associated with the period of living in darkness, characterized by obscurity about the cause and diagnosis of their mental health problems. This darkness period resulted in significant delays in the diagnoses. In terms of the cause, some participants associated the onset of mental health problems with supernatural causes such as the evil eye, witchcraft, or demonic possession. These participants seem to suggest that the onset of their symptoms coincided with a third party’s malevolent involvement. This supposed cause of distress, which differs from the medical model, has been previously documented in various populations [33, 34] including Arab-Islamic society [35–37]. This seems to indicate that belief in the supernatural cause of mental health problems persists across civilizations, income levels, and racial groups [38, 39]. On one hand, to conform to the biomedical model, it may be necessary to enhance public awareness about mental health problems through mass media, social media, and public lectures. On the other hand, traditional healing practices could be further scrutinized and integrated into existing healthcare practice. Such an integrative approach has been reported to have heuristic value in non-Western populations [40].

The second reason for living in darkness relates to the delayed diagnosis and treatment experienced by the AYA in part due to diagnostic overshadowing, or being unsure of the diagnosis. This delay may be partly explained by the fear and reluctance of families to seek mental health care, as reported among Arab populations [41]. It may also be, as in a US-based study of Asian Americans by Okazaki [42], that some families delayed seeking healthcare services because of the shame they felt and the stigma attached to mental health problems. In addition, our findings revealed that many physicians initially associated mental health problems with physical illness, which also lead to delays in treatment.

The present study and related literature appear to indicate that delayed diagnosis of mental health problems is a pervasive problem worldwide. For instance, a cross-sectional survey conducted in Vietnam concluded that people with severe mental health problems living in poor and middle-income countries often experience extended diagnosis delays, hindering their access to medical treatment [43]. Similarly, a UK study found that diagnostic overshadowing and the resulting delays were a significant issue often characterized by poor communication among key players [44]. All of these delays, unfortunately, lead to longer waiting times for diagnosis and treatment of the main pathology, with possibly serious consequences [45, 46]. These unhelpful delays and the lack of clarity on the actual cause of mental health problems among patients and families means that AYA continue to live in the dark for a prolonged period before they receive a proper diagnosis and commence treatment. This calls for evidence-based strategies to reduce the waiting period.

The perilous journey theme reveals three main challenges that face mentally ill AYA in the present cohort. The first is the short consultation time with their primary physicians and mental health workers. In Oman, healthcare services are provided free of charge to Omani citizens, creating significant strain on the limited mental health care resources available. This has been previously reported to adversely affect the doctor-patient relationship, as well as cause poor compliance which, in turn, can trigger poor health outcomes [47]. In a systematic review, Irving, Neves [48] scrutinized the literature from 1946 to 2016 on consultation time in primary healthcare settings around the world. The review identified 179 articles that fulfilled the inclusion criteria. It suggests that geography has a direct bearing on consultation time, with a clear-cut north-south divide; countries in the global south were more likely to have shorter consultations than those predominately in the global north. Countries in the global south are likely to fulfil the criteria of being lower- and middle-income countries [49], which often have a shortage of healthcare workers amid the rising tide of both communicable and non-communicable diseases. The role of geography might contribute to the observed short consultations in the present study, in a region where mental health services are understaffed and maldistributed [50]. In addition to the north-south divide, in any given society there is evidence to suggest that socio-economic status has a direct bearing on the consultation time. In France, Videau, Saliba-Serre [51] explored the consultation time of patients seeking help forMajor Depressive Disorder (MDD) from general practitioners. This study strongly suggested that consultation duration was invariably related to the socio-economic status; that is, the poor were given the shortest consultation time. In a close-knit society like Oman, one’s identity often owes its origin to the tribe. However, more recently, income is increasingly becoming a defining feature of social status [52]. Whether traditionally defined or modern finance-based socio-economic status may play a role in determining consultation time. Such short and rushed consultations added to the perilous nature of the journey as the patients felt not “being properly attended to” and described the visits as “non-productive”.

In addition, the psychotherapeutic sessions were not perceived by the interviewees to be constructive, for two possible reasons. First, many of the psychotherapy modalities dispensed by Omani psychologists rely on the Anglo-American model where there is emphasis on the individual as a separate entity. In Western psychology, when the ‘ideal self’ is incompatible with the ‘real self’, the individual is likely to experience guilt. Such societies have been labelled as guilt-based [53]. Prolonged intrapsychic guilt has the potential to trigger intrapsychic conflict which may culminate in full-blown mental health problems [54]. In contrast to guilt-based societies, interdependent Omani societies use shame as an instrument for socialization. Thus, individuals moulded in a collective mindset may find Western psychotherapy useless, as explained by one of the respondents. The point of divergence is that Western psychotherapy tries to unlock intrapsychic conflict [53], whereas in a shame-based society sources of stress are likely to be attributed to disequilibrium in social relationships. Thus, according to Al-Sinawi, Al-Adawi [55], when individuals experience stress and distress in Oman, they are likely to attribute their difficulty to an external agent such as “*hassad* (an evil spirit of contemptuous envy) or being targeted by *sihr* (sorcery)” (p. 697). Such attribution would require the therapist to disentangle social relationships rather than intrapsychic guilt. Therefore, it was not surprising that the respondent found psychotherapy “useless”. Secondly, the quality psychological interventions provided by clinicians has been criticized. According to Al-Darmaki and Yaaqeib [56], “The practice of providing psychological services such as counseling and psychotherapy remains loosely regulated by the authorities. The lack of enforced rules of practice have enabled ‘bogus’ psychologists and mental health workers who exploit legal loopholes to operate…” [56]. Related to this, it is a common observation that humanities graduates are employed to work as clinical psychologists [57]. To improve psychological outcomes, a concerted effort is needed to work on developing culture-sensitive intervention and, related to this, better quality assurance procedures for interventions provided by practitioners.

Another significant complication during the adolescents’ journey is the side effects of psychotropic medication. The participants reported untoward effects of the medication complicating their treatment. This drug-related distress seems to be a common problem across patient populations. For instance, Lucca, Varghese [58] revealed that adverse medication reactions range from 5.9% to 45% in the Indian psychiatry population. Similar drug-induced side effects were reported among the American population by Covell, Weissman [59]. Like our study, the most common distressing side effects were cognitive impairment, embarrassment from weight gain, dry mouth and sedation, and the feeling of being “weird” like a “zombie” [14, 60]. These findings point towards the need to improve the quality of physician consultations as well as mechanisms to reduce non-compliance with intervention due to side effects. One possible avenue is to explore adolescent-specific health education on the side effects of psychotropic medication [61]. In the Arabian Gulf region, pharmacotherapy appears to be the mainstay of intervention for people with mental distress [62, 63]. A concerted effort is needed to explore whether there is likely to be culture-specific psychosocial intervention that could be used as an adjunct to pharmacotherapy [64].

The third theme, uncertain future, relates to three fears: the possibility of relapse, the possibility of serious effects of the medication, and the possibility that patients may never recover well enough to return to normal functioning. These fears are deeply embedded in the psychological well-being of adolescents, affecting their adherence to treatment and, ultimately, their recovery. The psychological impact of these fears underlines the necessity of interventions that increase resilience and coping strategies among affected adolescents. The first uncertainty, possibility of relapse, is manifest in adolescents’ fear of again losing control of their thoughts. The literature describes the fear of relapse affecting not only patients, but extending to family members. A Canadian study showed family members of young people receiving services for a first-episode psychosis extremely worried about relapse, typically accompanied by considerable levels of dread and anxiety [65]. An earlier study reported that this is a serious condition that warrants attention since fear of recurrence has been linked to increased risk of relapse and poorer emotional recovery [66]. Furthermore, the study found that the greater the fear of relapse, the shorter was the duration before the actual relapse in people with mental health problems.

The second reason for the adolescent’s uncertainty relates to the side effects of medication. Such fears mean that they might not adhere to the regime, further complicating their recovery. A systematic evaluation of the factors for medication non-adherence in individuals with significant mental health problems found that side effects accounted for 27.8% of the reasons why patients quit taking medication. This is an important finding since interruption of treatment for as little as one day is associated with increased risk of hospitalization in patients with certain conditions such as schizophrenia [60, 67].

The third reason for uncertainty is the fear that the disease may compromise patients’ return to normal routines, such as school attendance. Usually, uncertainty increases when people are unable to plan for their future due to life-changing transformative conditions. The uncertainty the AYA experienced in our study is comparable to the uncertainty that was prevalent during the 2020–21 COVID-19 pandemic which significantly interfered with people’s social, economic and mental wellbeing [68]. As a remedy, such uncertainties call for strategies that increase the resilience and coping ability of affected AYA.

The lived experience of the AYA with mental health problems, pending further scrutiny, would require paradigm changes to meet their unmet needs. To address the concerns on living in darkness, concerted efforts are needed to reduce stigma and improve mental health literacy across all strata of society. Innovative approaches may include adding new content to the extant secondary and tertiary education curricula on mental health problems. In terms of how efforts to make the treatment journey less perilous, healthcare services in Oman should apply best practice in their prescription policy, as well as consider the development of culture-sensitive therapies. Health education is needed to enlighten AYA about the pros and cons of complying with the prescribed medication for mental health problems. As consultation time was deemed inadequate, sufficient interaction should be promoted. Last but not least, to address the concern of an uncertain future, deliberate efforts should be employed to help educate AYA on prognostic indicators of their presenting mental health problems. The lifestyle changes that lead to positive prognostic indicators should be emphasized. In the final analysis, the new mantra should be to employ a biopsychosocial approach to safeguard the wellbeing of AYA.

### Limitations

While the small sample size might be seen as a limitation in the current study, the idiographic nature of the present qualitative research would imply more in-depth information was obtained, with a focus on the vertical rather than the horizontal. Additionally, the number of participants in the present study is consistent with other qualitative studies [30, 69]. A possible limitation of the study is that some participants were interviewed in a hospital setting. While every effort was made to ensure participants felt comfortable during the interview, the hospital setting may have affected their openness to our inquiries, leading to demand characteristics [70]. The themes that emerged in the present study, however, appear to discount the presence of demand characteristics.

## Conclusions

The present study provides new insights into the experience of Omani AYA with mental health problems. The AYA interviewed faced several challenges, the most significant of which was lack of understanding of their condition at the onset of symptoms, leading to delays in the treatment process. Our findings indicate that there is a need for developing culture-sensitive interventions and improving the quality of psychological services in Oman. Implications of our findings lie in the development of approaches and mechanisms for preventing or mitigating difficulties faced by AYA with mental health problems.
